# Short-Term Effects of Spironolactone/Hydrochlorothiazide on Respiratory Support in Preterm Infants with Bronchopulmonary Dysplasia: A Retrospective Before–After Study

**DOI:** 10.3390/jcm15062096

**Published:** 2026-03-10

**Authors:** Osman Selçuk Duysak, Murat Konak, Muhammed Yaşar Kılınç, Saime Sündüs Uygun, Hanifi Soylu

**Affiliations:** Division of Neonatology, Department of Pediatrics, Selçuklu Medical Faculty, Selçuk University, Konya 42000, Turkey; drosmanselcukduysak@gmail.com (O.S.D.); drmuhammed007@gmail.com (M.Y.K.); uygunsaime@hotmail.com (S.S.U.); hasoylu@hotmail.com (H.S.)

**Keywords:** bronchopulmonary dysplasia, preterm infant, diuretics, spironolactone, hydrochlorothiazide, oxygen requirement

## Abstract

**Objectives:** Diuretics are frequently used in bronchopulmonary dysplasia (BPD), yet evidence describing their short-term physiological effects remains limited. This study aimed to describe early changes in respiratory support parameters and safety outcomes following combined oral spironolactone and hydrochlorothiazide (SP/HCTZ) therapy in preterm infants with BPD. **Methods**: A retrospective, single-center before–after observational study was conducted. Preterm infants diagnosed with BPD who initiated SP/HCTZ therapy were included. Respiratory parameters (FiO_2_, PEEP, and flow rate) and serum electrolytes were compared between Day 1 (initiation) and Day 3 of treatment. A predefined clinical response was defined as either a ≥10% reduction in FiO_2_ or a step-down in respiratory support modality. **Results:** Fifty-six infants (mean gestational age 27.7 ± 2.3 weeks) were analyzed. After 72 h of SP/HCTZ therapy, mean FiO_2_ decreased from 26.2 ± 6.3% to 22.4 ± 3.4% (*p* < 0.001). Significant reductions were also observed in PEEP and cannula flow rates (*p* = 0.004 and *p* = 0.003, respectively). Overall, 39 infants (69.6%) met the predefined clinical response criteria. The prevalence of hyponatremia (Na < 133 mmol/L) increased from 7.1% at baseline to 25.0% on Day 3 (*p* = 0.039). **Conclusions**: Initiation of SP/HCTZ was temporally associated with short-term reductions in respiratory support parameters; however, these findings should be interpreted as associations rather than treatment effects. Given the increased frequency of hyponatremia by Day 3, close electrolyte monitoring appears warranted during the early phase of therapy.

## 1. Introduction

Bronchopulmonary dysplasia (BPD) remains a major morbidity among very preterm infants and is characterized by impaired lung development and prolonged need for respiratory support. Diuretics are frequently used in established or evolving BPD with the rationale of reducing pulmonary interstitial edema and improving pulmonary mechanics; however, evidence supporting routine or prolonged diuretic therapy is limited and practice varies widely. Combination therapy with a thiazide and spironolactone has been evaluated in small trials and is still used in many neonatal intensive care units, but contemporary real-world data describing short-term respiratory parameter changes and safety signals remain limited [1,2,3].

Spironolactone is a potassium-sparing diuretic and competitive aldosterone receptor antagonist acting at the distal nephron, where it reduces sodium reabsorption while limiting potassium loss. Hydrochlorothiazide is a thiazide diuretic that inhibits the sodium–chloride cotransporter in the distal convoluted tubule, thereby promoting natriuresis and mild diuresis. In combination, these agents may enhance sodium excretion while partially mitigating potassium depletion, with the theoretical potential to reduce pulmonary interstitial fluid and improve lung compliance in selected infants with BPD [4,5,6,7,8].

Randomized trials of oral distal-tubule diuretics (thiazides with or without spironolactone) in infants with established BPD have reported mixed short-term improvements in pulmonary mechanics and FiO_2_, but no consistent reduction in duration of oxygen therapy or other longer-term outcomes [4,5,6,7,8,9]. Loop diuretics such as furosemide may acutely improve airway resistance, yet are associated with electrolyte and acid–base disturbances, including hypochloremic metabolic alkalosis [10,11,12,13]. Lung ultrasound has also been explored as a bedside tool to quantify pulmonary edema and monitor response to diuretic therapy [14]. Despite continued use in clinical practice, uncertainty remains regarding the short-term physiologic benefit and early safety profile of these regimens in contemporary NICU populations [9,15].

The aim of this study was to evaluate short-term (72 h) changes in respiratory support parameters and selected safety outcomes following initiation of spironolactone/hydrochlorothiazide (SP/HCTZ) therapy in preterm infants with BPD.

## 2. Materials and Methods

### 2.1. Study Design and Setting

This was a single-center, retrospective, before–after observational study conducted in a tertiary neonatal intensive care unit (NICU). The study evaluated short-term changes in respiratory support parameters and selected laboratory safety outcomes between treatment Day 1 (initiation of SP/HCTZ) and treatment Day 3 (approximately 72 h after initiation). The study period covered patients treated between January 2019 and December 2021. Clinical data were routinely recorded as part of standard care, and data extraction and statistical analyses were performed only after institutional ethics approval was granted.

In our unit, oxygen therapy is titrated to maintain peripheral oxygen saturation (SpO_2_) within a target range of 90–95% used in routine NICU practice, and FiO_2_ is adjusted accordingly.

### 2.2. Participants

Eligible participants were preterm infants diagnosed with BPD who received combined SP/HCTZ therapy during hospitalization.

Inclusion criteria were as follows:

1. Diagnosis of evolving or established BPD;
2. Initiation of SP/HCTZ therapy during NICU stay;
3. Availability of paired FiO_2_ measurements on both treatment Day 1 and Day 3.

Exclusion criteria were as follows:

1. Major congenital anomalies expected to substantially affect respiratory outcomes;
2. Absence of paired Day 1 and Day 3 data for the relevant variable, in which case infants were excluded from that specific analysis.

### 2.3. Exposure: SP/HCTZ Regimen

Initiation of SP/HCTZ therapy was based on clinical assessment of persistent oxygen requirement and suspected pulmonary fluid burden in infants with evolving or established BPD, according to unit practice and attending physician judgment.

The dosing regimen used in this study (spironolactone 2 mg/kg/day plus hydrochlorothiazide 20 mg/kg/day, enteral, divided twice daily) was administered according to the unit’s established protocol for an initial short-term therapeutic trial.

Although some infants may have had prior diuretic exposure before the evaluated 72 h window, no additional diuretics or systemic corticosteroids were administered during the defined study period.

### 2.4. Definitions

BPD severity at 36 weeks’ postmenstrual age (PMA) was graded using the respir-atory-support-based Jensen et al. system (Grade 1–3) [3], consistent with contemporary NICHD recommendations. Respiratory support categories were defined as invasive ventilation (intubated), continuous positive airway pressure (CPAP), high-flow nasal cannula (HFNC; flow ≥ 2 L/min), and low-flow nasal cannula (LFNC; flow < 2 L/min).

Respiratory support at SP/HCTZ initiation was coded as an ordinal category:

1 = invasive ventilation (intubated);
2 = CPAP;
3 = HFNC ≥ 2 L/min;
4 = LFNC < 2 L/min.

Hyponatremia was defined as serum sodium (Na) < 133 mmol/L.

İnspired gas flow rate refers to blended gas flow delivered via nasal cannula systems, allowing titration of FiO_2_ rather than administration of pure oxygen.

### 2.5. Outcomes

The primary outcome was the paired change in FiO_2_ (%) from treatment Day 1 to Day 3.

Secondary respiratory outcomes included paired changes in PEEP/CPAP level, mean distending pressure, peak inspiratory pressure (PIP), and inspired gas flow rate (L/min), when paired data were available.

A predefined short-term clinical response was defined a priori as either:

1. A ≥10% relative reduction in FiO_2_ from Day 1 to Day 3; and/or
2. A step-down of at least one respiratory support category by Day 3.

The 72 h evaluation window was selected to reflect routine clinical practice in our unit, where early response to SP/HCTZ is typically assessed within the first 3 days. In infants without apparent early clinical benefit, continuation of therapy is generally reconsidered in order to avoid unnecessary drug exposure and potential adverse effects, whereas treatment is usually continued in those with apparent benefit.

Safety outcomes included paired changes in serum sodium, potassium, creatinine, albumin, and bicarbonate (HCO_3_^−^) between Day 1 and Day 3, as well as paired change in hyponatremia status.

Body weight on treatment Day 1 and Day 3 was available and evaluated as an additional descriptive parameter. In addition, infants continued to receive adjunctive caffeine therapy during the evaluated 72 h period according to routine NICU practice. Because caffeine treatment was ongoing rather than newly initiated during this timeframe, it was not analyzed as a separate exposure variable.

### 2.6. Data Source and Availability

Clinical and laboratory data were extracted retrospectively from medical records. De-identified individual participant data are available from the corresponding author upon reasonable request, subject to institutional approval and applicable privacy restrictions.

### 2.7. Data Quality Procedures and Sensitivity Analyses

Because retrospective datasets may contain implausible laboratory values due to transcription or documentation inconsistencies, prespecified plausibility checks were applied for sensitivity analyses. Values outside plausible physiological ranges were considered implausible and excluded only in sensitivity analyses (albumin <1.0 or >6.0 g/dL; potassium <2.0 or >7.0 mmol/L). Primary analyses were performed on all available paired data; sensitivity analyses repeated the paired comparisons after excluding implausible values.

Missing data were handled by complete-case paired analysis for each outcome, and no imputation was performed.

Longer-term outcomes such as total duration of diuretic therapy and oxygen supplementation were beyond the predefined scope of this short-term analysis.

### 2.8. Statistical Analysis

Statistical analyses were performed using IBM SPSS Statistics 25 (IBM Corp., Ar-monk, NY, USA). Data distribution was assessed using the Shapiro–Wilk test and visual inspection of histograms. Continuous variables are presented as mean ± standard deviation (SD) or median (interquartile range, IQR), as appropriate. Paired comparisons between Day 1 (initiation) and Day 3 values were performed using the paired *t*-test for normally distributed variables and the Wilcoxon signed-rank test for non-normally distributed variables. Categorical variables are expressed as frequencies and percentages. Paired categorical outcomes (e.g., presence of hyponatremia at Day 1 versus Day 3) were analyzed using the McNemar test. When expected cell counts were less than five, the exact McNemar test was applied. Missing data were handled by complete-case paired analysis for each outcome; no imputation was performed. Respiratory support categories were coded as ordinal variables for descriptive purposes: invasive mechanical ventilation, CPAP, HFNC, and LFNC.

All tests were two-sided, and a *p*-value <0.05 was considered statistically significant.

### 2.9. Ethics and Reporting

The study was conducted according to the guidelines of the Declaration of Helsinki and approved by the Selçuk University Faculty of Medicine Non-Interventional Clinical Research Ethics Committee (Approval No.: 2022/214; 26 April 2022). Informed consent was waived due to the retrospective design and use of de-identified data. Patient consent was waived due to the retrospective design and use of de-identified clinical data. The study is reported in accordance with STROBE recommendations for observational studies.

### 2.10. Use of Generative Artificial Intelligence (GenAI)

Generative AI (ChatGPT, OpenAI; version GPT-5.2) was used only to assist with language refinement and clarity; it was not used for study design, data collection, analysis, interpretation, or figure generation.

## 3. Results

### 3.1. Baseline Characteristics at SP/HCTZ Initiation

A total of 56 preterm infants with BPD who received SP/HCTZ and had paired primary outcome data were included. Baseline characteristics at SP/HCTZ initiation are summarized in Table 1. The mean gestational age was 27.7 ± 2.3 weeks, the mean birth weight was 1035 ± 351 g, and the mean postmenstrual age at treatment initiation was 34.8 ± 1.6 weeks. Respiratory support modality at treatment initiation and BPD severity are detailed in Table 1.

### 3.2. Changes in Respiratory Support Parameters (Day 1 vs. Day 3)

**Figure 1:**
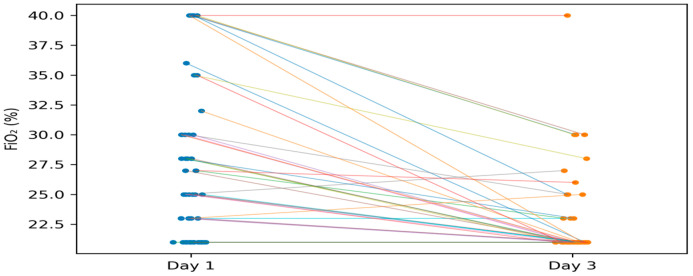
Paired FiO_2_ (%) values on treatment Day 1 (initiation) and Day 3 (~72 h) in 56 infants. Each line represents one infant. *p* value from paired comparison (Wilcoxon signed-rank test).

The primary analysis focused on paired 72 h changes in FiO_2_ in the full cohort, whereas secondary respiratory and subgroup analyses were restricted to infants with modality-specific paired data. The primary outcome was the paired change in FiO_2_ over 72 h in all 56 infants. FiO_2_ decreased from 26.23 ± 6.32% on Day 1 to 22.46 ± 3.45% on Day 3 (mean absolute change: −3.77 percentage points, *p* < 0.001). Figure 1 shows individual paired FiO_2_ trajectories.

Secondary ventilatory parameters were available only for infants receiving the relevant mode of respiratory support and with paired Day 1 and Day 3 documentation; therefore, these analyses were conducted in subsets. Among infants with available paired measurements, PEEP/CPAP level decreased from 7.56 ± 1.76 to 6.30 ± 2.92 cmH_2_O (*n* = 27, *p* = 0.004), mean distending pressure decreased from 9.27 ± 3.94 to 8.06 ± 4.91 cmH_2_O (*n* = 26, *p* = 0.027), and inspired gas flow decreased from 3.25 ± 2.10 to 1.96 ± 2.45 L/min (*n* = 26, *p* = 0.003). PIP did not change significantly (20.40 ± 7.52 to 19.90 ± 8.56 cmH_2_O, *n* = 10, *p* = 0.878).

According to the predefined composite criteria, 39 infants (69.6%) met the definition of predefined short-term clinical response. However, this composite outcome should be interpreted cautiously, particularly in infants with lower baseline FiO_2_ values, where relative reductions may overestimate perceived clinical benefit.

### 3.3. Laboratory Values and Safety Outcomes (Day 1 vs. Day 3)

Paired laboratory changes are summarized in Table 2. Bicarbonate (HCO_3_^−^) increased from 24.86 ± 3.44 to 26.82 ± 3.35 mmol/L (*n* = 56, *p* < 0.001). Serum sodium decreased from 136.77 ± 3.99 to 134.11 ± 7.05 mmol/L (*n* = 56, *p* = 0.006), and the proportion of infants with hyponatremia (Na < 133 mmol/L) increased from 4/56 (7.1%) on Day 1 to 14/56 (25.0%) on Day 3 (*p* = 0.039). Potassium showed no significant change (*p* = 0.430), and creatinine showed a non-significant decrease (*p* = 0.057). Albumin increased from 3.03 ± 0.57 to 3.74 ± 2.61 g/dL (*p* < 0.001), although the marked variability in Day 3 values should be interpreted cautiously.

Body weight was also evaluated as an additional descriptive parameter. In the overall cohort, body weight increased slightly from 1664.6 ± 648.8 g on Day 1 to 1678.5 ± 658.1 g on Day 3, but this change was not statistically significant (*p* = 0.074).

### 3.4. Subgroup Analysis by Respiratory Modality at Treatment Initiation (IMV vs. Non-Invasive)

At treatment initiation, 9 infants were receiving IMV and 47 infants were on non-invasive support (CPAP/HFNC/LFNC). FiO_2_ decreased in both groups over 72 h (IMV: 33.33 ± 7.23% to 25.44 ± 6.33%, Wilcoxon *p* = 0.021; non-invasive: 24.87 ± 5.19% to 21.89 ± 2.28%, Wilcoxon *p* < 0.001). The magnitude of FiO_2_ change differed between groups (ΔFiO_2_ Day 3–Day 1: −7.89 ± 7.36 vs. −2.98 ± 4.01; Mann–Whitney U *p* = 0.045), although this comparison should be interpreted as exploratory given the small IMV subgroup.

Hyponatremia (Na < 133 mmol/L) increased from Day 1 to Day 3 in both groups (IMV: 22.2% to 33.3%, McNemar exact *p* = 1.000; non-invasive: 4.3% to 23.4%, McNemar exact *p* = 0.012). Day 3 hyponatremia prevalence did not significantly differ between groups (Fisher’s exact *p* = 0.676). Body weight also did not change significantly over 72 h in either group (IMV: 1516.1 ± 936.0 g to 1535.6 ± 947.7 g, *p* = 0.426; non-invasive: 1693.0 ± 554.5 g to 1705.9 ± 552.6 g, *p* = 0.131). Full subgroup results are provided in Table S1 in the Supplementary Materials.

## 4. Discussion

In this retrospective before–after study of preterm infants with BPD, initiation of SP/HCTZ was temporally associated with a short-term reduction in oxygen requirement over 72 h. Among infants with available paired measurements, PEEP/CPAP level, mean distending pressure, and oxygen flow also decreased, suggesting that a subset experienced early improvement in respiratory support needs following diuretic initiation.

Our results are directionally consistent with earlier randomized, crossover, and controlled trials of thiazide-based regimens, with or without spironolactone, in infants with BPD/chronic lung disease, which primarily reported improvements in pulmonary mechanics and/or short-term respiratory parameters [4,5,6,7,8]. However, the broader literature also emphasizes uncertainty regarding whether short-term physiologic changes translate into durable, clinically meaningful outcomes and highlights heterogeneity across trials and practice patterns [9,15]. The predefined ≥10% relative FiO_2_ reduction threshold was selected a priori as a pragmatic marker of clinically noticeable short-term change. However, we acknowledge that relative reductions at lower baseline FiO_2_ values may overestimate perceived clinical benefit; therefore, this composite outcome should be interpreted cautiously.

It is also important to recognize that much of the evidence supporting diuretic use in infants with BPD originates from an earlier era of neonatal intensive care and differs substantially from current practice. Changes in ventilation strategies, oxygen targeting, routine adjunctive therapies, nutritional support, and evolving definitions of BPD may limit the direct applicability of older studies to contemporary NICU populations. In this context, the most recent systematic review suggests that although diuretics may be associated with short-term physiologic improvement in selected infants, the overall level of evidence remains limited with respect to longer-term outcomes such as duration of oxygen therapy, length of hospital stay, and other durable clinical endpoints [9]. Accordingly, diuretic responsiveness in BPD is likely heterogeneous and influenced by baseline lung fluid burden, ventilatory strategy, evolving disease trajectory, and co-morbidities.

The physiologic rationale for benefit is plausible. Reduction of pulmonary interstitial fluid and improved lung compliance may translate into lower oxygen needs in selected infants, particularly when pulmonary edema contributes meaningfully to respiratory burden. Lung ultrasound-based assessments may help contextualize fluid-related phenotypes in future work [14]. However, lung ultrasound was not used as part of the present study protocol, and no standardized imaging-based method was available to systematically document changes in pulmonary fluid burden. Therefore, mechanistic interpretation of fluid-related changes remains limited.

Safety signals were notable. Serum sodium decreased over the 72 h window and the proportion of infants meeting criteria for hyponatremia increased by Day 3. These findings support close electrolyte monitoring when initiating SP/HCTZ in preterm infants with BPD. Prior neonatal literature has highlighted electrolyte and acid–base disturbances associated with diuretic exposure [11], and more recent data in infants with BPD suggest that diuretic exposure patterns are associated with differences in serum electrolytes [13]. The observed increase in hyponatremia is biologically plausible given the natriuretic effect of thiazide diuretics, which increase distal tubular sodium excretion. Dilutional mechanisms related to fluid management strategies may also have contributed [13]. However, because detailed fluid balance, sodium supplementation, and enteral intake data were not consistently available in this retrospective dataset, mechanistic interpretation remains limited. The marked variability in Day 3 albumin values should likewise be interpreted cautiously. As retrospective datasets may contain occasional implausible laboratory values or documentation inconsistencies, prespecified plausibility checks were applied in sensitivity analyses.

Our findings should also be interpreted within the broader context of real-world practice. Despite limited evidence supporting long-term benefit, diuretic use in BPD has historically been common and variable across centers [15,16]. This discordance between evidence-based certainty and clinical practice is increasingly recognized. More recent implementation studies suggest a shift away from routine chronic diuretic use through guideline-driven practice change, reflecting attempts to better align clinical care with evolving evidence and safety considerations [17].

Strengths of this study include paired within-subject comparisons over a narrow 72 h timeframe, which helps reduce between-infant confounding, and reporting of parameter-specific sample sizes for paired measurements. The narrow evaluation window was also clinically relevant in our setting, because early response to SP/HCTZ was typically assessed within the first 3 days of therapy and continuation of treatment was generally reconsidered in infants without apparent early benefit in order to avoid unnecessary drug exposure and potential adverse effects. However, this design does not capture longer-term efficacy.

Limitations include the retrospective, single-center design; absence of a concurrent control group; and potential residual confounding by concurrent clinical changes and regression to the mean. Accordingly, the observed changes should be interpreted as temporal associations rather than direct treatment effects, and causality cannot be inferred. Secondary ventilatory parameters should also be interpreted cautiously. Because these variables were only available for infants receiving the relevant mode of respiratory support and with paired Day 1 and Day 3 documentation, these analyses were necessarily restricted to subsets of the cohort. This may have introduced selection bias and limits the generalizability of these secondary findings. Follow-up was short, and longer-term outcomes such as duration of respiratory support, total duration of oxygen supplementation, and discharge respiratory status were not assessed. Because SP/HCTZ was administered as a combined regimen, the individual contributions of spironolactone and hydrochlorothiazide cannot be distinguished. To address data-quality concerns, we prespecified plausibility checks and repeated key analyses after excluding implausible outliers; results for the primary outcome were unchanged.

The dosing regimen used in our unit (spironolactone 2 mg/kg/day plus hydrochlorothiazide 20 mg/kg/day, enteral, divided twice daily) should be interpreted as a pragmatic institutional protocol rather than a standardized evidence-based regimen. Future studies should incorporate controlled designs, standardized dosing and monitoring protocols, and longer follow-up to determine whether early changes translate into clinically meaningful benefits, to identify responder phenotypes, and to better characterize the risk–benefit profile of SP/HCTZ in this population [9,15].

## 5. Conclusions

Among preterm infants with BPD, initiation of SP/HCTZ was temporally associated with short-term reductions in FiO_2_ over 72 h, but also with an increased frequency of hyponatremia. Given the retrospective before–after design and lack of a control group, these findings should be interpreted as associations rather than evidence of treatment efficacy. Prospective controlled studies are needed to define clinical effectiveness, identify responder phenotypes, and optimize safety monitoring.

## Figures and Tables

**Table 1:** Baseline characteristics at SP/HCTZ initiation.

Characteristic	Value
Gestational age, weeks	27.7 ± 2.3
Birth weight, g	1035 ± 351
Male sex, *n* (%)	36 (64.3)
IUGR, *n* (%)	4 (7.1)
Postmenstrual age at SP/HCTZ initiation, weeks	34.8 ± 1.6
BPD grade at 36 weeks’ PMA (3), *n* (%)	Grade 1: 5 (8.9%); Grade 2: 23 (41.1%); Grade 3: 28 (50.0%)
Respiratory support at initiation, *n* (%)	Intubated: 9 (16.1%); CPAP: 19 (33.9%); HFNC ≥ 2 L/min: 23 (41.1%); LFNC < 2 L/min: 5 (8.9%)
Predefined short-term clinical response, *n* (%)	39 (69.6)

Abbreviations: BPD, bronchopulmonary dysplasia; CPAP, continuous positive airway pressure; HFNC, high-flow nasal cannula; IUGR, intrauterine growth restriction; LFNC, low-flow nasal cannula; PMA, postmenstrual age; SP/HCTZ, spironolactone/hydrochlorothiazide.

**Table 2:** Changes in respiratory parameters and laboratory values (Day 1 vs. Day 3).

Variable	*n*	Day 1	Day 3	Change	*p*-Value
FiO_2_ (%)	56	26.23 ± 6.32	22.46 ± 3.45	−3.77	<0.001
PEEP/CPAP level (cmH_2_O)	27	7.56 ± 1.76	6.30 ± 2.92	−1.26	0.004
Mean distending pressure (cmH_2_O)	26	9.27 ± 3.94	8.06 ± 4.91	−1.21	0.027
PIP (cmH_2_O)	10	20.40 ± 7.52	19.90 ± 8.56	−0.50	0.878
Flow (L/min)	26	3.25 ± 2.10	1.96 ± 2.45	−1.29	0.003
HCO_3_ (mmol/L)	56	24.86 ± 3.44	26.82 ± 3.35	1.96	<0.001
Sodium (mmol/L)	56	136.77 ± 3.99	134.11 ± 7.05	−2.66	0.006
Hyponatremia (Na < 133), *n* (%)	56	4 (7.1)	14 (25.0)	+17.9 pp	0.039
Potassium (mmol/L)	56	4.52 ± 0.79	4.46 ± 0.65	−0.06	0.430
Albumin (g/dL)	56	3.03 ± 0.57	3.74 ± 2.61	0.71	<0.001
Creatinine (mg/dL)	56	0.49 ± 0.46	0.38 ± 0.20	−0.11	0.057

Values are mean ± SD unless stated otherwise. Δ = Day 3 − Day 1. n represents infants with paired Day 1 and Day 3 values for the specified parameter. PEEP/CPAP level includes invasive PEEP (IMV) and CPAP pressure levels. Mean distending pressure includes IMV mean airway pressure and documented CPAP mean pressure/derived distending pressure when available. PIP was obtained from invasive mechanical ventilator settings only. For hyponatremia, change is reported as percentage-points (pp). Flow refers to blended gas flow delivered via nasal cannula systems.

## Data Availability

De-identified data are available from the corresponding author upon reasonable request, subject to institutional approvals; the data are not publicly available due to privacy and confidentiality restrictions.

## Supplementary Materials

The following supporting information can be downloaded at: https://www.mdpi.com/article/10.3390/jcm15062096/s1, Table S1: Subgroup analysis (IMV vs. Non-invasive).

## Abbreviations

The following abbreviations are used in this manuscript:

BPD	Bronchopulmonary Dysplasia
PEEP	Positive end-expiratory pressure
PIP	Peak İnspiratory Pressure
HFNC	High-Flow Nasal Cannula
LFNC	Low-Flow Nasal Cannula
CPAP	Continuous Positive Airway Pressure
