# Supplementary material for: Short-Term Effects of Spironolactone/Hydrochlorothiazide on Respiratory Support in Preterm Infants with Bronchopulmonary Dysplasia: A Retrospective Before–After Study
Source: J Clin Med. 2026 Mar 10;15(6):2096. doi: 10.3390/jcm15062096 (PMC13026276; doi:10.3390/jcm15062096)
Supplement: Supplementary file 1 [file jcm-15-02096-s001.zip › jcm-4177177-supplementary.pdf]

Table S1. Subgroup analysis (IMV vs Non-invasive)

Outcome	IMV (n=9) Day1	IMV Day3	IMVΔ(D3–D1)	p (within)	Non-invasive Day 1	Non-invasive Day 3	Non-invasive Δ (D3–D1)	p (within)	p (between Δ)
FiO_2 (%)	33.33±7.23	25.44± 6.33	−7.89±7.36	0.021	24.87±5.19	21.89±2.28	−2.98±4.01	<0.001	0.045
Sodium (mmol/L)	135.78±8.24	134.00± 6.24	−1.78±9.15	0.498	136.96±2.65	134.13±7.26	−2.83±7.56	0.007	0.991
Hyponatremia (Na <133), n (%)	2 (22.2)	3 (33.3)	—	1.000†	2 (4.3)	11 (23.4)	—	0.012†	0.676‡
Weight (g)	1516.11±935.97	1535.56± 947.68	+19.44±51.20	0.426	1693.02±554.48	1705.89±552.64	+12.87±62.30	0.131	0.902
Flow (L/min)	NA	NA	NA	NA	3.25±2.10 (n=26)	1.96±2.45 (n=26)	−1.29±2.02	0.002	NA
PEEP/CPAP level (cmH_2O)	8.89±1.69	8.33±2.00	−0.56±1.13	0.157	6.89±1.41 (n=18)	5.28±2.80 (n=18)	−1.61±2.33	0.011	0.277
Mean distending pressure (cmH_2O)	13.67±3.32	12.17±4.00	−1.50±2.72	0.093	NA	NA	NA	NA	NA
PIP (cmH_2O)	21.78±6.50	18.78±8.26	−3.00±6.56	0.136	NA	NA	NA	NA	NA

Footnotes: Values are mean ± SD. Δ = Day 3 − Day 1. p(within): Wilcoxon signed-rank. p(between Δ): Mann–Whitney U. McNemar exact was used for paired categorical comparisons; Fisher's exact was used for Day 3 prevalence by group. PEEP/CPAP level includes invasive PEEP and CPAP pressure levels. Mean distending pressure includes IMV mean airway pressure and documented CPAP mean pressure/derived distending pressure when available. PIP was obtained from invasive mechanical ventilator settings only. Flow refers to blended gas flow via nasal cannula systems.
